# Dexamethasone-Loaded Bioactive Coatings on Medical Grade Stainless Steel Promote Osteointegration

**DOI:** 10.3390/pharmaceutics13040568

**Published:** 2021-04-16

**Authors:** Jan Rožanc, Marko Žižek, Marko Milojević, Uroš Maver, Matjaž Finšgar

**Affiliations:** 1 Faculty of Medicine, Institute of Biomedical Sciences, University of Maribor, 2000 Maribor, Slovenia; jan.rozanc@um.si (J.R.); marko.milojevic@um.si (M.M.); 2 Faculty of Medicine, University of Maribor, 2000 Maribor, Slovenia; 3 Faculty of Chemistry and Chemical Engineering, University of Maribor, 2000 Maribor, Slovenia; marko.zizek@student.um.si

**Keywords:** dexamethasone, carboxymethyl cellulose, medical grade stainless steel, controlled drug release, electrochemistry

## Abstract

In this study, a multilayer bioactive coating based on carboxymethyl cellulose (CMC) and dexamethasone (DEX) was prepared on medical-grade stainless steel (AISI 316LVM). Its aim was the controlled drug delivery of the incorporated anti‑inflammatory drug, which at the same time promotes osteogenic differentiation of mesenchymal stem cells. Due to DEX’s limited solubility in physiological fluids, which limits the loading capacity of coatings, it was further combined with β-cyclodextrin to increase its concentration in the bioactive coating. Controlled release of DEX from the multilayer coating was achieved in four steps: a “burst”, i.e., very fast, release step (in an immersion interval of 0–10 min), a fast release step (10–30 min), a slow-release step (60–360 min), and a plateau step (360–4320 min), following a zero-order release or Higuchi model release mechanism. Successful layer-by-layer coating formation was confirmed using attenuated total reflectance Fourier transform infrared spectroscopy (ATR-FTIR). It was shown that the application of the coating significantly increases the hydrophilic character of AISI 316LVM, and also significantly increases the surface roughness, which is known to promote cell growth. In addition, electrochemical measurements demonstrated that the coating application does not increase the susceptibility of medical-grade stainless steel to corrosion. In vitro cell testing using all cell types with which such coatings come into contact in the body (osteoblasts, chondrocytes, and mesenchymal stem cells (MSCs)) showed very good biocompatibility towards all of the mentioned cells. It further confirmed that the coatings promoted MSCs osteogenic differentiation, which is the desired mode of action for orthopedic implants.

## 1. Introduction

By 2050, the proportion of the world’s population aged 60 years and over will nearly double from 12% (in 2015) to 22%. Of cause for concern is not only the total expected number of elderly persons but also the pace at which the population is ageing. According to the World Health Organization (Fact Sheets, Aging and Health), every single country is facing major challenges in ensuring that its health and social systems will survive this demographic shift unharmed [1].

An important aspect of aging is the accumulation of various molecular and cellular damage over time, which leads to a gradual decrease in physical (and mental) capacity [2]. Among the most critical consequences are diseases of the musculoskeletal system, including several bone‑related diseases [3]. Osteoporosis, osteoarthritis, and others, often necessitate clinical interventions, including bone replacement (i.e., the implantation of artificial materials) [4].

Implantations have been part of regular clinical treatment for centuries. Despite this fact, the range of base materials is far from vast. Medical grade stainless steel (AISI 316LVM) is still among the most commonly used implant materials due to its low cost and other suitable properties (e.g., mechanical stability over time, corrosion resistance, biocompatibility) [5,6,7]. Nevertheless, none of the mentioned properties is optimal, especially considering the high life expectancy in the western world and the relatively low age at which such implantations (e.g., hip replacements) might occur. Namely, stability over 20 years is challenging for the most sophisticated of the current implants [8]. This is even truer for substrates such as AISI 316LVM with the above-mentioned long-term instabilities [9].

An increasingly interesting approach to improving AISI 316LVM lies in its functionalization with coatings intended to prolong its lifespan [10,11], increase fast osteointegration [12], and/or provide improved long-term corrosion resistance [13,14]. Various materials have been employed for this purpose (e.g., selenium nanoparticles [15], hydroxyapatite in various forms [16,17], polylactic acid [18], peptide coatings [19], etc.). However, it seems that most of the interest lies in biocompatible coatings based on polysaccharides, such as alginate (ALG), carboxymethyl cellulose (CMC), and others [12,20,21,22,23].

These and other studies report various approaches to coating preparation. However, the trend should be to ease the process. In contrast, the focus should be shifted towards improving their osteointegration potential (i.e., the promotion of osteogenic differentiation, osteoblast growth, and biomineralization) and/or towards coating stability, allowing for use in all kinds of implantations [24,25,26,27].

Dexamethasone (DEX) is a commonly applied glucocorticoid with a vast range of systemic pharmacodynamic effects. Among others, glucocorticoids are labeled anti-inflammatory drugs in their purest essence, alleviating all levels of inflammation in the body. As part of systemic therapy, they are used to treat various inflammatory diseases [28] and the application of implants [29,30]. Furthermore, DEX was shown to boost osteogenic differentiation in a variety of studies [31,32]. Both of the mentioned effects, the multi-fold anti-inflammatory activity and osteogenic differentiation, make DEX an interesting drug to incorporate in coatings on metal implants in orthopedic applications.

However, the use of DEX and other glucocorticoids does not come without problems. Among these, its low solubility in water renders its inclusion in formulations harder than expected [33]. Due to the latter, solutions for its functionalisation were developed, including combinations of different polymers [33], the use of solubilizing agents [34,35], and the preparation of formulations based on “bimodal” materials with hydrophilic and lipophilic parts (e.g., cyclodextrins (CDs)) [36]. Although the literature reports some examples of DEX combinations, to the best of our knowledge, our study is the first to combine β-cyclodextrin (β-CD) with DEX to prepare coatings on metal implants for orthopedic applications.

This work reports on the development and characterization of new bioactive and biocompatible coatings on AISI 316LVM. The coatings consisted of carboxymethylcellulose (CMC) and dexamethasone (DEX). A multilayer coating of alternating CMC and DEX layers was developed to provide a bioactive therapeutic treatment platform for an anti-inflammatory effect and simultaneous osteogenic differentiation. Both effects are achieved by successfully manipulating the coating composition (including the concentration of DEX) and drug release mechanisms. Detailed characterization of the coating was performed by attenuated total reflectance Fourier transform infrared spectroscopy (ATR-FTIR), contact angle measurements, and atomic force microscopy (AFM). In addition, the corrosion susceptibility of AISI 316LVM before and after the application of these coatings was checked by electrochemical impedance spectroscopy (EIS) and cyclic polarization measurements (CP). Finally, the coatings’ functional properties were determined by evaluating their in vitro release performance and cell testing using human tissue-based cells that come into contact with orthopedic implants. These included mesenchymal stem cells (MSCs), osteoblasts, and chondrocytes.

## 2. Experimental

### 2.1. Sample Preparation

The AISI 316LVM samples were cut out from a 2-mm-thick plate in the shape of discs with a diameter of 15 mm. The discs were ground on a rotating grounding device with 320 and 500-grit SiC papers (Struers, Ballerup, Denmark) under a water stream until the surface layer was removed and unidirectional scratches were visible. The grinding leftovers and any possible fatty residues were removed by immersion in an ultrasonic bath for 5 min. This bath contained 50 vol.% ethanol/50 vol.% ultrapure water. Ultrapure water, with a resistivity of 18.2 MΩ cm at 25.0 °C, was obtained with the Milli-Q^®^ system, supplied by EMD Merck KGaA, Darmstadt, Germany. As-prepared substrates were passivated for 1 h in 30 wt.% nitric acid (purity ≥ 65%, Sigma Aldrich, St. Louis, MO, USA), rinsed with ultrapure water, and dried under a stream of air. Electrochemical measurements were performed in 0.9 wt.% solution of NaCl (NaCl was pro analysis, supplied by Carlo Erba Reagents, Italy). A solution of 0.9 wt.% NaCl was employed as it is the main compound in body fluid, which causes corrosion.

### 2.2. Solutions and Coatings Preparation

CMC solution (containing 0.5 wt.% CMC) was prepared using CMC (low viscosity, Sigma Aldrich, St. Louis, MO, USA) and ultrapure water. As the solubility of DEX in water is very low (89 mg/L at 25 °C) [33,37], increased solubility can be achieved with the addition of β-CD [38,39]. β-CD with a purity ≥97% was used for this purpose (Sigma Aldrich). The mass concentration ratio of DEX:β-CD was 1:8. The mentioned ratio was chosen based on the solubility data of DEX and β-CD from the literature [40], as well as considering the fact that in the solution with this ratio, all DEX, even at a concentration of 2.5 mg/mL, was possible to dissolve. At a higher concentration of β-CD an undissolved residue remained in the solution.

Two DEX solutions were prepared, i.e., solutions named DEX1 (containing 1 mg/mL DEX; Sigma‑Aldrich, Darmstadt, Germany) and DEX2.5 (containing 2.5 mg/mL DEX). Both solutions were prepared in ultrapure water and placed for 5 min in an ultrasonic bath.

β-CD and DEX were weighed into separate glass flasks. To prepare the β-CD solution, a flask was halfway filled with ultrapure water. The weighed β-CD amount was transferred into the flask, followed by adding the weighed DEX amount. The flask was filled with ultrapure water to the top of the flask’s neck. Despite extensive mixing, some sediment was still present. For this purpose, the flask was placed in an ultrasonic bath for 5 min (the procedure was repeated, if necessary). When there was no visible sediment left in the flask, the flask was filled up to the mark. The solution was then filtered into glass containers with lids (using syringes with filter nozzles).

**Figure 1:**
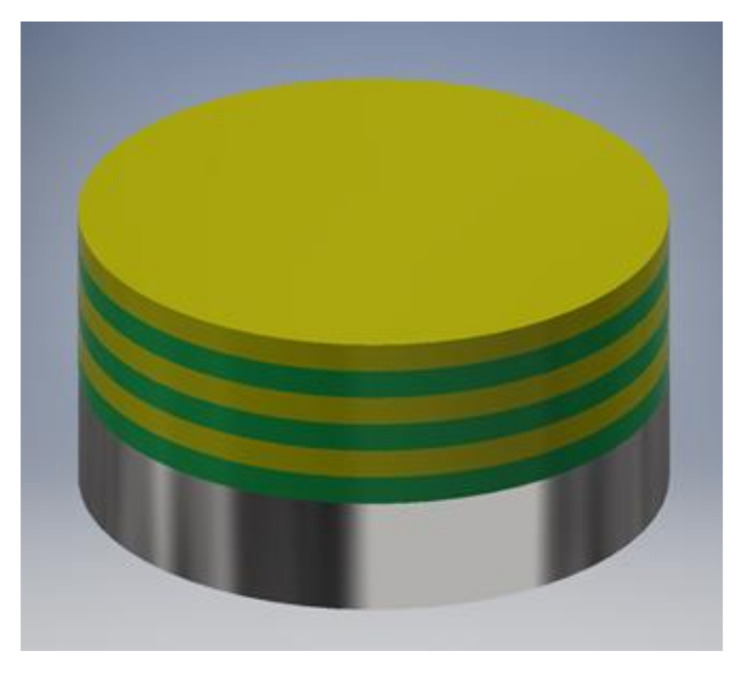
Representation of the multilayer coating design (the green layer is CMC and the yellow layer is DEX solubilised with β-CD).

As-prepared AISI 316LVM samples (as described in Section 2.1) were placed in the center of a POLOS SPIN-150i-NPP spin coater (SPS Vertriebs GmbH, Germany) and held in place with a vacuum pump. To ensure even spreading of the first layer, 200 µL of CMC solution was placed in the center of the substrate surface, and the sample was spun. The spinning parameters were the same for every layer application: spinning velocity 2500 rpm, acceleration 500 rpm/s, and spinning time 180 s. 100 µL of the DEX solution (either DEX1 or DEX2.5) was placed in the center of the CMC-coated substrate to apply the second layer. Next, an additional CMC layer was applied by placing 100 µL of CMC solution on the previously coated surface. A multilayer coating was formed on the substrate by alternately adding the CMC and the DEX layers, as schematically depicted in Figure 1. Coated samples were named 3CMCxDEXy, where 3 and x are the number of layers and y is the concentration of the DEX solution. Depending on the DEX solution used, the samples were named 3CMC3DEX1, 3CMC2DEX1, 3CMC3DEX2.5, and 3CMC2DEX2.5, where the DEX1 and DEX2.5 terms stand for the solution used to prepare these coatings (as described above).

### 2.3. Coating Characterisation

The alternating multilayer coating formation was investigated by means of ATR-FTIR measurements using an Agilent Cary 630 FTIR spectrometer with a diamond ATR module (Agilent Technologies, Santa Clara, CA, USA). ATR-FTIR measurements were performed in the 4000–630 cm^−1^ wavenumber range.

Contact angle (*CA*) measurements were performed with an OCA 200 device (DataPhysics Instruments GmbH, Filderstadt, Germany). A 1 µL droplet of ultrapure water was placed on a sample, and the droplet was photographed. The image was then processed with SCA software (DataPhysics Instruments GmbH, Filderstadt, Germany). The contact angle was determined as the angle between the sample surface and the droplet’s tangential border line at the three-phase contact point [41].

An evaluation of the topography properties and roughness parameters of the coated samples was performed by AFM in tapping mode. A Keysight Technologies 7500 AFM device was used (Keysight Technologies, Santa Barbara, CA, USA). Before acquiring images, the samples were dried under a stream of dry high-grade (99.999 vol.%) nitrogen. The scanning was performed using ATEC-NC-20 silicon cantilevers (Nanosensors, Germany) with a force constant of 12–110 N/m and a resonance frequency of 210–490 kHz. The imaging was performed at room temperature. Each sample was scanned at a size of 10 × 10 µm^2^ and 1 × 1 µm^2^ with a resolution of 2048 × 2048 pixels [6]. The corresponding roughness parameters were calculated using PicoImage software (Keysight Technologies, Santa Barbara, CA, USA) [6].

### 2.4. In Vitro Drug Release

An Automated Transdermal Diffusion Cells Sampling System (Logan System 912-6, Somerset, NJ, USA) was used to analyze the drug release behavior. The coated samples were placed in Franz diffusion cells, filled with phosphate-buffered saline—PBS (0.137 M NaCl, 0.01 M phosphate buffer, and 0.0027 M KCl; pH 7.4 at 25 °C). PBS tablets (Sigma-Aldrich, Germany) were used to prepare these solutions. The coated side of the immersed sample was facing up. The temperature during the experiments was kept stable at 37 °C. The magnetic bar for stirring the medium during the experiment (50 rpm) was placed below the diffusion cell. Samples of 1 mL were collected in different intervals (1 min, 5 min, 10 min, 20 min, 30 min, 60 min, 120 min, 180 min, 240 min, 300 min, 360 min, 1440 min, 2880 min, and 4320 min). The volume of the collected samples was replaced with fresh PBS at 37 °C. The concentration of released DEX was measured by UV/VIS spectrophotometry (Cary 60 UV-Visible Spectrophotometer, Agilent, Waldbronn, Germany) at a wavelength of 241 nm. The dilution effect from sample withdrawal and media replacement was accounted for in the concentration calculations using the Beer-Lambert law. To determine how many different release stages are present, the first derivative of the cumulative mass (d(*m*_norm_/*m*_max_)/d*t*) was calculated and plotted, where *m*_norm_ and *m*_max_ stand for the mass of all released DEX until a certain time point, and the total released/incorporated DEX, respectively, while *t* stands for the release time. The release kinetics were subsequently tested with commonly applied mathematical models for drug release. These included zero order, first order, Hixson–Crowel, Higuchi, and Korsmeyer–Peppas models [12].

### 2.5. Electrochemical Measurements

All electrochemical measurements were performed in a 0.9 wt.% NaCl solution, in a glass electrochemical cell at 37 °C (controlled by a thermostat), and were executed using Autolab PGSTAT204 (Metrohm Autolab, Utrecht, Holland). A saturated calomel electrode (SCE) and a graphite rod were used for reference and counter electrodes, respectively. The working electrodes were uncoated and coated samples inserted in a Teflon holder, sealed with an o-ring. The area of the working electrode exposed to the solution was 1 cm^2^.

Chronopotentiometry, EIS, and CP curve measurements were performed in sequence, starting with chronopotentiometry the moment after sample immersion in the solution for up to 1 h of immersion. Then an EIS measurement was performed. Afterwards, a chronopotentiometry measurement for up to 3 h of immersion was performed. This procedure was repeated until EIS measurements were collected after 1, 3, 5, 7, 10, 15, 20, 24, 36, 48, 60, and 72 h of immersion. Finally, a CP measurement was performed after 72 h of immersion.

EIS measurements were performed at the open circuit potential (*E*_oc_) in the 5 mHz–1 MHz frequency range using a 10 mV peak‑to‑peak amplitude. The obtained EIS data were fitted with ZView2 software (Scribner Associates Inc., Southern Pines, NC, USA). CP measurements started at −0.250 V vs. *E*_oc_ potential and a potential sweep rate of 0.1 mV/s in the anodic direction. When the current density (*i*) reached a value of 1 mA/cm^2^, the potential sweep was reversed in the cathodic direction until the starting potential was reached.

### 2.6. In Vitro Cell Response

Three different cell types were used to assess the in vitro response. Human adipose-derived mesenchymal stem cells (hADMSC) (PCS-500-011, ATCC, Botolph Claydon, UK) (passages 2–4) were grown in mesenchymal stem cell basal medium supplemented with an MSC growth kit (ATCC, UK) and 1 wt.% penicillin/streptomycin. Human bone tissue-derived osteoblasts (CRL-11372, ATCC, UK) were grown in Advanced ADMEM supplemented with 5 wt.% FBS and 1 wt.% penicillin/streptomycin. Human articular chondrocytes were isolated and characterized as described previously [42] and grown in Advanced ADMEM supplemented with 5 wt.% FBS and 1 wt.% penicillin/streptomycin.

For observation of the cell morphology, the cells were seeded on the coated and uncoated AISI 316LVM samples in a 24-well plate at a density of 4·10^4^ cells/well and incubated for 48 h. Phalloidin (1000× Phalloidin stock solution in dimethyl sulfoxide DMSO (Abcam, UK), 1/1000 dilution in PBS with 1 wt.% bovine serum albumin (BSA; Sigma-Aldrich, Munich, Germany), and 0.1 wt.% Tween 20 (Sigma-Aldrich, Munich, Germany)) was used to stain for actin filaments. After 90 min of incubation at room temperature and in a dark room, the cells were washed, and Fluoroshield Mounting Medium with 4′,6-diamidino-2-phenylindole DAPI was used to stain the nuclei. The cells were visualized using a fluorescent microscope (EVOS FL, Thermo Fisher Scientific, Waltham, MA, USA) at suitable wavelengths for the respectively used dyes (excitation/emission: DAPI = 306/460 nm and Phalloidin = 556/574 nm).

To determine cell viability, a tetrazolium salt MTT (3(4,5 dimethylthiazolyl-2)-2,5-diphenyltetrazolium bromide) assay was used (Sigma-Aldrich, Steinheim, Germany). Briefly, cells were seeded onto the prepared experimental and control substrates at a density of 4·10^4^ cells/well in a 24-well plate. After 3 and 6 days of incubation at 37 °C and 5 wt.% CO_2_, the medium was removed and the cells were washed with PBS, followed by the addition of 250 µl of MTT assay solution and incubated for 2 h at 37 °C. 100 μL aliquots of the culture solution from each sample were then transferred to a 96-well plate and the absorbance was measured spectrophotometrically at 570 nm using a Varioskan multiplate reader (ThermoFisher, Dreieich, Germany).

In order to assess the osteogenic and chondrogenic differentiation potential of MSCs on CMC/DEX‑coated substrates, the expression of osteogenic (collagen type I (COLIA1) and osteocalcin (OCN)) and chondrogenic (collagen type II (COLIIA1) and aggrecan (AGR)) protein markers were evaluated. Briefly, cells were seeded onto the samples placed in a 24-well plate at a concentration of 2.5 × 10^4^ cells/well and incubated for 14 and 21 days. The medium was changed once a week. After incubation, the cells were fixed with a Fixation solution (Merck KGaA, Darmstadt, Germany) and the following antibodies were used for immunostaining to assess the expression of the protein markers: Rb pAb anti-COLIA1 (Abcam, Cambridge, UK), Rb pAb anti-COLIIA1 (Abcam, Cambridge, UK), Ms mAb anti-AGR (Abcam, Cambridge, UK), Goat pAb to Rb—AF 594 (Abcam, Cambridge, UK), Rb pAb to Ms—AF 488 (Abcam, Cambridge, UK), and Rb pAb anti-OCN-AF488 (Bioss, Woburn, MA, USA).

## 3. Results and Discussion

Novel materials intended for biomedical applications require a series of analyses to understand their properties in a targeted environment and to assess their safety and efficiency in contact with cells at the target site in the body [43,44]. Since an anti‑inflammatory and osteogenesis-boosting coating for “long-term” orthopedic applications was developed (e.g., hip replacements), most of the tests performed were oriented towards controlling the main coating composition, long-term stability, drug release performance, and cell response with tissue-specific target cells.

### 3.1. Multilayer Structure Characterisation

**Figure 2:**
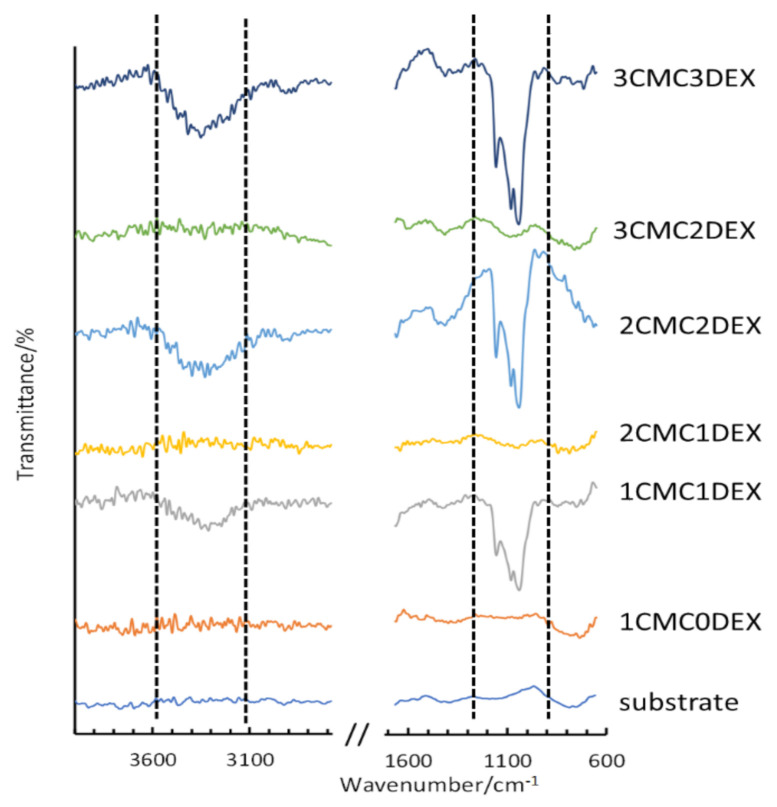
ATR-FTIR measurements on AISI 316LVM after successive application of alternating CMC and DEX layers.

To confirm the multilayer structure of the coating on the AISI 316LVM, ATR-FTIR measurements were performed for the respective DEX and CMC layers. Figure 2 shows the spectra for the uncoated AISI 316LVM and all successive layers (from bottom to top), forming the final 3CMC3DEX coating. Each application of a DEX coating layer results in the formation of an intense ATR-FTIR peak (marked within the dashed lines shown in Figure 2). After adding a CMC layer, the characteristic peaks for DEX (almost completely) disappear, indicating that the CMC coating effectively covered the DEX layer. These ATR-FTIR spectra, therefore, show the successful layer-by-layer build-up of the multilayer CMC-DEX coating. Furthermore, a comparison of the spectra for all the components included in the coatings (CMC, DEX, and β-CD) is shown in Figure S1 (Supplementary Materials). An overlay of the spectra for DEX, β-CD, and their mixed solution is shown in Figure S2 (Supplementary Materials).

### 3.2. Hydrophobicity Measurements

The contact angles were measured for the uncoated and coated samples. The results are given in Table 1. For all coated samples, the contact angle decreased significantly, indicating the hydrophilic nature of the main polymer component (CMC) of the coatings. When DEX was in the top-most layer (a six-layer coating), the contact angle dropped slightly compared to the coating with CMC as the top-most layer (a five-layer coating); however, this change is not significant. Furthermore, another conclusion can be made based on these results. Namely, the insignificant changes in hydrophilicity between the samples with DEX and CMC as the top-most layer, respectively, show that, despite the alternating CMC/DEX structure being confirmed using ATR-FTIR, the main coating character is nevertheless defined by the polymer. The latter plays an important part in the coating’s observed dissolution behavior, where surface wetting importantly contributes to successful control of the drug release (see below, Section 3.3).

### 3.3. Coating Topography and Morphology Evaluation Using AFM

**Figure 3:**
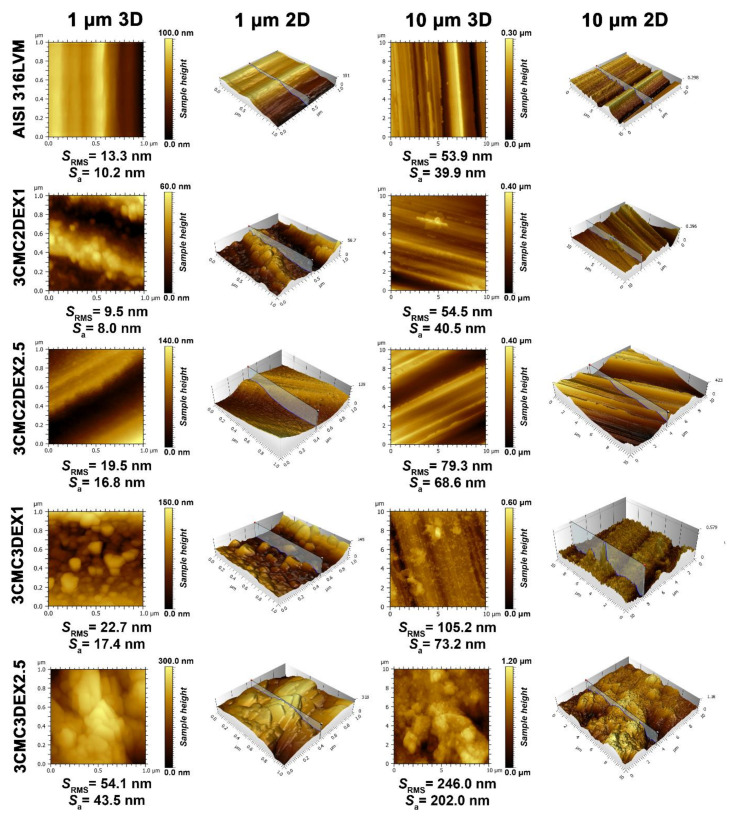
AFM topography measurements for all samples and AISI 316LVM in 2D and 3D, accompanied by the corresponding roughness parameters.

In order to evaluate the influence of the prepared coatings on the substrate topography and roughness, AFM was used on two scan sizes (10 × 10 and 1 × 1 µm^2^) (Figure 3). Upon first examination, successful coating application and, with it, significantly changed surface features (i.e., topography), are immediately evident. The parallel running surface features of the substrate (introduced through grinding, as described in the Experimental section) are evident for all samples, except for the final sample, i.e., 3CMC3DEX2.5, whose top-most layer was loaded with the higher drug concentration. While the first applied coating with the lower drug concentration (3CMC2DEX1) did not significantly increase the surface roughness, the subsequent samples gradually increased the surface roughness in an expected pattern. Namely, by increasing the number of applied layers and increasing the drug concentration the roughness parameters increase. This is also evident from the obtained AFM images (showing the obtained sample topographies). One interesting phenomenon observed is the presence of “sphere-like” shapes starting with the first applied coating (3CMC2DEX1), which disappear in the final sample (3CMC3DEX2.5), where these surface features seem to have “coalesced” into bigger structures. The appearance of these bigger structures also led to a clear jump in the roughness parameters (comparing this sample to any of the others). In contrast to one of our previous studies, in which CMC was used as part of a coating with a different drug-loaded [12], here no smooth surface can be observed. In the present study, the drug-polymer coatings seem to have formed a different kind of organization, which leads to increased surface roughness compared to the mentioned study and very different topography. The latter includes surface features beyond the ones formed by grinding. An interesting result is also that despite the increased roughness due to the increased DEX concentration (when comparing the samples with CMC as the top-most layer, i.e., 3CMC2DEX1 and 3CMC2DEX2.5), the topography seems to be “smoother”, as shown in the AFM images of the samples with a higher DEX concentration. Furthermore, in the case of samples with DEX as the top-most layer, the roughness, and observable topography indicate the same trend.

Similar observations can be made based on the concrete measured roughness parameters, which confirms that adding a DEX layer at the top and increasing the DEX concentration in the coatings increases the surface roughness. Such an increased surface roughness favours cell attachment and is an important improvement of the base substrate properties in terms of applicability as part of orthopaedic implants [45,46].

### 3.4. In Vitro Drug Release

**Figure 4:**
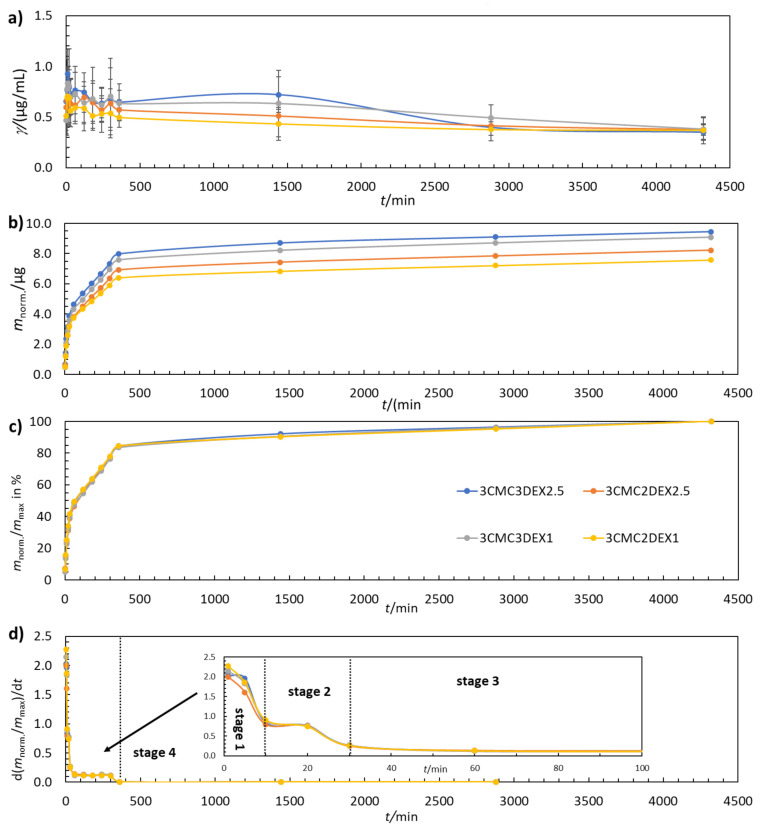
(**a**) The change in DEX concentration during drug release testing, (**b**) the cumulative mass of the released DEX, (**c**), the normalised mass of the released DEX, and (**d**) the first derivative of the released amount of DEX for coated samples. The error bars in (**a**) represent 95% confidence intervals.

Controlled in vitro drug release is a much-desired property of multilayer coatings in almost any biomedical application. In this study, the coatings were examined by evaluating their drug release performance from various perspectives. Figure 4a shows that the DEX concentration increases and decreases with increasing *t*. Such behavior fits well with the multilayer coating structure, where CMC and DEX layers are present in an alternating manner. The maximum amount of released DEX was determined by plotting the cumulative mass of DEX release over time (Figure 4b). As expected, the additional DEX layer in the six-layer coating (3CMC3DEX1 and 3CMC3DEX2.5) leads to higher cumulative DEX release compared to the five-layer coating (3CMC2DEX1 and 3CMC2DEX2.5). Figure 4c shows that all four coatings follow the same four-stage drug release mechanism (the separation of the first three stages is shown in Figure 4d, which presents the first derivative of the release profiles given in Figure 4c). Stage 1 (0–10 min) represents a “burst” release stage in which high amounts of DEX are released almost immediately (in clinical terms, this leads to rapid achievement of the therapeutic drug concentration). Stage 2 (10–30 min) represents a “fast” release stage, where a high release rate of DEX is still achieved, but more slowly than in the “burst” stage (in clinical terms, this still increases the drug concentration and contributes to the maximal drug concentration). In stage 3 (60–360 min) the release rate slows down and remains constant over time (which means that the drug concentration starts to fall slowly yet remains in a concentration interval that maintains the therapeutic effect). The last stage, stage 4 (360–4320 min), is the so-called “plateau” stage in which the release rate is the slowest, and the remaining DEX is released from the coating (the concentration slowly falls, and another dose would be required in a clinical setting during this stage).

#### Mathematical Modelling of the Drug Release Stages

All identified release stages in Figure 4 were fitted using five common mathematical release models by converting the data into the respective parameters given in Table 2. In the table, *w* is the mass fraction of released DEX, *Q*_max_ (%) is the maximum amount of released DEX, and *Q*_t_ (%) is the amount of released DEX at a given *t*. These parameters were plotted as required to fit the data with respective release models in order to obtain a linear trend line. The latter was then used to obtain the square of the correlation coefficient (*R*^2^). The release model with the highest *R*^2^ value, i.e., for which the *R*^2^ value was closest to 1.000, was considered to be the most probable to explain the release mechanism for the respective stage. The obtained *R*^2^ values are given in Table 3.

Both DEX1-containing coatings, i.e., 3CMC3DCF1 and 3CMC2DCF1, follow the zero-order release model in all four steps. This means that the release kinetics are not affected by the drug concentration. Coatings containing DEX2.5 (3CMC3DCF2.5 and 3CMC2DCF2.5) follow the zero-order model in stages 1 and 3, while in stage 2 they follow the Higuchi model. The Higuchi model describes drug release as a diffusion-controlled process. The model is based on Fick’s law, depending on the square root of *t*. Considering the latter, it seems that the coatings with a higher DEX load achieved internal saturation of the dissolved drug, which limited the maximal drug release from 3CMC3DCF2.5 and 3CMC2DCF2.5. In the coatings with a lower DEX load (3CMC3DCF1 and 3CMC2DCF1), the internal concentration in the coatings was not high enough to limit the overall release rate. From a practical perspective regarding the application of such coatings, the incorporated concentration of DEX should be less than the 2.5 mg/mL used to allow for more straightforward control over patient-tailored drug release performance. In stage 4, 3CMC3DEX2.5 continues to follow the Higuchi model, while 3CMC2DEX2.5 returns to zero‑order model release.

### 3.5. Long-Term Stability—The Corrosion Susceptibility of the Coated Samples

Electrochemical measurements were carried out to check whether the applied coatings significantly influenced the susceptibility to corrosion of the AISI 316LVM samples. Corrosion susceptibility was investigated in a 0.9 wt.% NaCl solution, which is the most important corrosive medium in body fluids. The tests were carried out at 37 °C to simulate the conditions in the human body. At least three replicate measurements were performed, followed by an EIS fitting procedure. The obtained fitted parameters for the replicate measurements were checked for possible outliers using the Grubbs’ test [47], and if detected they were discarded. The reported values were average values calculated with at least three values without outliers.

**Figure 5:**
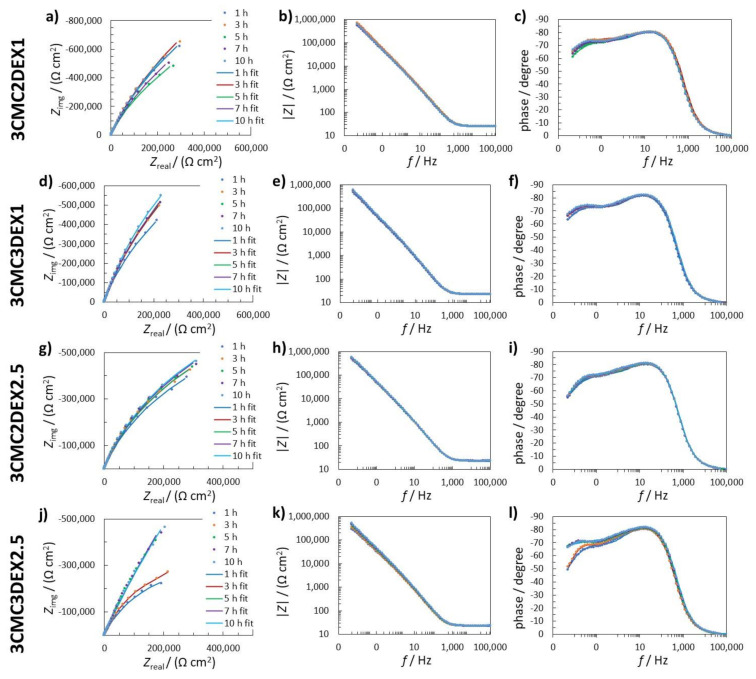
(**a**,**d**,**g**,**j**) Nyquist impedance; (**b**,**e**,**h**,**k**) Bode impedance; and (**c**,**f**,**i**,**l**) phase angle EIS spectra measured in 0.9 wt.% NaCl solution for (**a**–**c**) 3CMC2DEX1; (**d**–**f**) 3CMC3DEX1; (**g**–**i**) 3CMC3DEX1; and (**j**–**l**) 3CMC3DEX1 samples. The measured data are represented with dots, whereas fitted data are given as solid lines.

Figure 5 shows measured and fitted EIS data for the coated samples (one of three replicate measurements is given for each system). In order to explain the electrochemical mechanism of the coated samples in 0.9 wt.% NaCl solution, several possible equivalent electrical circuits (EEC) were used to fit the EIS response. The goodness of the fitting procedure was evaluated with the c^2^ value [48], i.e., the lower the c^2^, the better the obtained fit, and the probability that the EEC employed is accurate is higher. The EEC with the lowest c^2^ was *R*_Ω_(*Q*_2_(*R*_2_(*Q*_1_*R*_1_))) (*R* and *Q* stand for the resistance and constant phase element, respectively). *R*_Ω_ represents the uncompensated resistance. The element *Q* in this case represents the capacitance *C*, according to *C*_x_ = (*R*_x_*Q*_x_)^1/*n*x^/*R*_x_ [49,50,51]. The impedance of CPE is *Z*(CPE) = (*Q*(j*w*)*^n^*)^–1^, where the symbols *Z*, j, *w*, and *n* represent the impedance, imaginary unit (j^2^ = 1), angular frequency, and CPE power, respectively. In the present fitting procedure, *n* was close to 1, confirming that *Q* represents *C*.

The first time constant (*Q*_1_*R*_1_) denotes the electrochemical properties of the double-layer capacitance (represented by *Q*_1_) and the charge transfer resistance (represented by *R*_1_). The second time constant (*Q*_2_*R*_2_) represents the surface layer properties, resulting from the combination of the oxide layer and the coating (the time constants representing the oxide and the coating were too close to be separated with the EIS fitting procedure). The sum of all resistances, apart from *R*_Ω_, represents the value of the polarisation resistance *R*_p_, which characterizes how the metallic material resists the transfer of electrons to the electroactive species in solution. The higher the *R*_p,_ the higher the resistance of the metallic material to general (uniform) corrosion.

**Figure 6:**
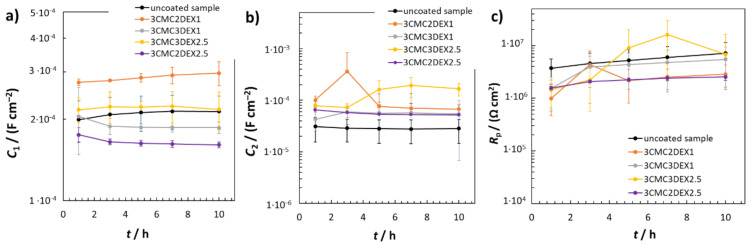
EIS fitted data for uncoated and coated samples; (**a**) *C*_1_, (**b**) *C*_2_, and (**c**) *R*_p_ fitted values obtained using *R*_Ω_(*Q*_2_(*R*_2_(*Q*_1_*R*_1_))) EEC.

Figure 6 shows the EIS fitted data obtained by using *R*_Ω_(*Q*_2_(*R*_2_(*Q*_1_*R*_1_))) EEC. The average *C*_1_ values were similar for the uncoated sample and the coated samples with 6 layers, i.e., 3CMC3DEX1 and 3CMC3DEX2.5 (Figure 6a). On the other hand, the coated samples with 5 layers showed different *C*_1_ properties than the uncoated sample. The 3CMC2DEX1-coated sample had higher and the 3CMC2DEX2.5-coated sample lower average *C*_1_ values compared with the uncoated sample. As expected, the surface layer properties (the average capacitance values *C*_2_) were different for the coated samples compared to the uncoated sample, i.e., for every coated sample, the average *C*_2_ values were higher than that for the uncoated sample. The latter is a consequence of the coating application. The most important parameter obtained from the EIS fitting procedure is the *R*_p_ value. The average *R*_p_ values obtained for the coated samples are shown in Figure 6c (the average *R*_p_ values for the uncoated samples are given for comparison [48]). The change in *R*_p_ values for the coated samples and uncoated sample is not significant (as tested with the ANOVA with a 95% confidence level). Therefore, the coatings applied have no significant influence on the general corrosion susceptibility of AISI 316LVM.

**Figure 7:**
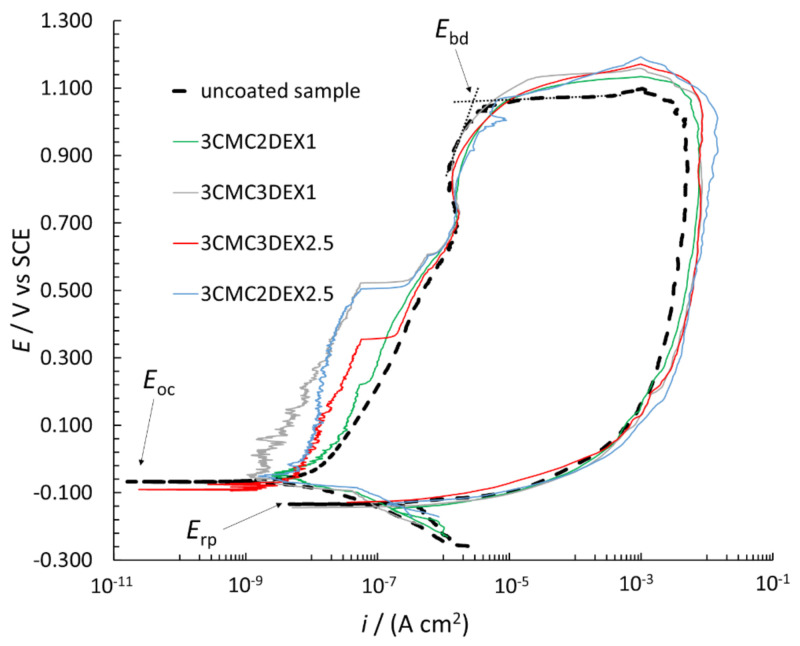
CP curves measured for uncoated and coated AISI 316LVM in 0.9 wt.% NaCl solution.

In order to further investigate the possible increased localized corrosion susceptibility of the coated samples compared to the uncoated sample, CP curve measurements were performed (Figure 7). The breakdown potential (*E*_bd_), repassivation potential (*E*_rp_), and *E*_oc_ are designated in the figure. The *E*_bd_ is determined by extrapolation (as shown in the figure), with the *i* suddenly increasing due to the surface breakdown, indicating significant localized corrosion (pitting and crevice formation). The *E*_rp_ defines the potential, based on which evaluation of the repassivation capability is possible, i.e., if *E*_rp_ is at more positive potentials than *E*_oc_, the metallic material can repassivate. However, none of the measured samples in Figure 7 has that capability (neither the uncoated nor coated samples). Moreover, the *E*_rp_ is similar for all samples. All CP curves are in the cathodic region (in the potential region from −0.250 V vs. *E*_oc_ to the *E*_oc_) at similar *i*. On the other hand, the CP curves in the anodic region (more positive than *E*_oc_) up to approximately 0.500 V vs. SCE are at different *i* for the uncoated and coated samples, i.e., the curves are at lower *i* for the coated compared to the uncoated samples. The latter indicates that these coatings even mitigate corrosion of AISI 316LVM to a certain degree. Moreover, the *E*_bd_ is at similar values for all CP curves, indicating similar localized corrosion behavior for both the uncoated and coated samples. After the breakdown in the reverse scan, the *i* was higher than that for the forward scan, which is typical of stainless steel materials in chloride solution (once corrosion starts, it can no longer be prevented). Based on the above explanations, the CP curve measurements suggested that the susceptibility to corrosion of the samples with the applied coatings did not increase, but minor corrosion protection of the coated samples can be expected.

### 3.6. The In Vitro Effect on Cell Viability, Morphology, and Differentiation

**Figure 8:**
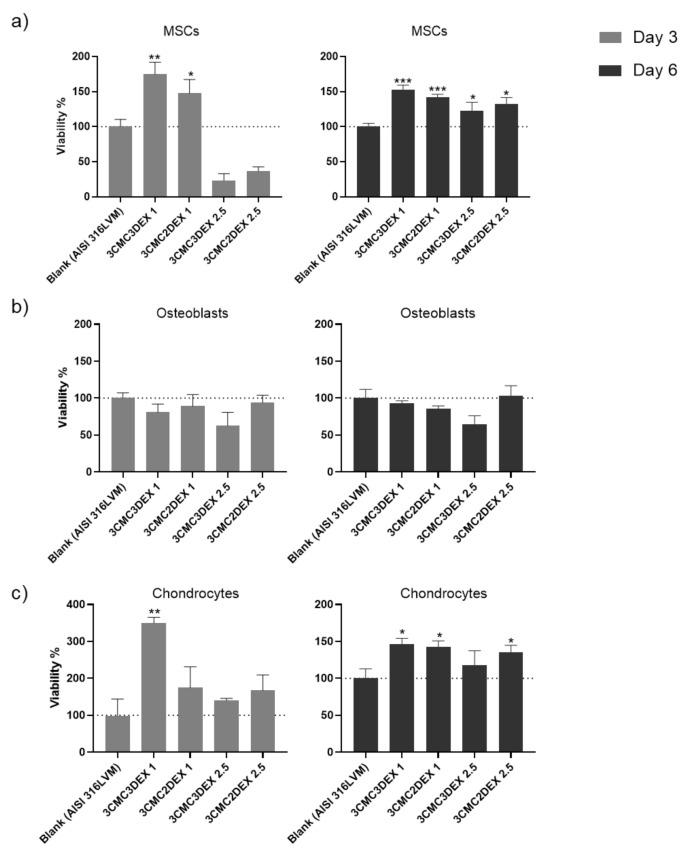
The cell viability testing results of (**a**) mesenchymal stem cells, (**b**) osteoblasts, and (**c**) chondrocytes cultured on uncoated and CMC/DEX-coated AlSI 316LVM scaffolds after 3 and 6 days of incubation. Cell viability is expressed as % relative to control (uncoated AISI 316LVM). Values are shown as the mean ± standard deviation (three replicates). Statistical significance was defined as * *p* < 0.05, ** *p* < 0.005, and *** *p* < 0.0005 compared to the control sample (ANOVA test).

In order to assess the biocompatibility of the CMC/DEX-coated samples, the cell viability was first analyzed to check if it is affected after exposure to the materials. Such biocompatibility testing is crucial in developing biomedical materials since it is directly connected with their safety and efficacy. For example, it can indicate possible toxic effects during exposure to a specific target tissue, rendering such materials inappropriate for use. A direct contact test was performed for this purpose, whereas all three main cell types in the target tissue (mesenchymal stem cells—MSCs, osteoblasts, and chondrocytes) were used for testing. As the direct contact test demands, the cells were seeded directly onto the coated and uncoated (control) substrates to assess their metabolic activity (MTT assay) after 3 and 6 days as an indirect indicator of cell viability. The results of the cell viability testing are shown in Figure 8. A significant increase in MSC cell viability on all samples compared to control was observed after 6 days (Figure 8a). Similarly, viability increased already after 3 days, except for the samples with a higher DEX concentration (containing 2.5 mg/mL DEX), indicating the unfavorable growth effects of high DEX concentrations at the site of immediate contact with the MSCs.

When assessing the viability of the osteoblasts (Figure 8b), similar growth potential on coated and uncoated substrates was observed except for 3CMC3DEX2.5, which seems to have inhibited cell viability. The effects were damped throughout by the CMC layer on 3CMC2DEX2.5, which indicated that the top layer should be either the polymer (CMC) or a layer containing a lower DEX concentration.

Lastly, it was evident that chondrocytes displayed better growth potential on almost all of the coated surfaces, with the lowest values observed for the 3CMC3DEX2.5 sample and the highest values (compared to the control) observed for the 3CMC3DEX1 sample. These results point to a dose-sensitive response to DEX. Considering that all of the cells in this study are of human origin, the obtained results indicate that the prepared samples are safe for use in orthopedic applications.

The cells’ cytoskeleton (actin) was stained and analyzed with fluorescent microscopy to further analyze the cellular morphology and adhesion. The analysis showed that all of the tested cell types adhered well on all the substrates. However, they displayed various shapes, including different distinct appearances, when grown on CMC or DEX as the direct contact layer. It appears that MSCs grown on the substrate with DEX as the top layer display a more elongated, fibroblast-like morphology. In contrast, cells grown on CMC display a more triangular or star-shaped morphology. Mesenchymal stem cells (MSCs) in general possess a fibroblast-like cell shape, which to some degree enables them to differentiate [52]. Therefore, such a shape is expected for these cells. On the other hand, the star‑shaped features observed on coatings with CMC as the top-most layer were previously reported to be the so-called “rapidly self-renewing cells” [53]. Although the differences in the measured viabilities for the cells grown on different coatings (using the MTT assay) are rather small, these nevertheless seem to be in agreement with this definition.

The osteoblasts displayed a round, cuboid-like morphology, except for the cells on 3CMC3DEX2.5, where the DEX concentration affected overall cell growth. Apart from the mentioned sample, there were no significantly different morphological features observed between the other three samples regardless whether CMC or DEX was the top layer.

The analysis of the chondrocytes showed flattened or disc-shaped cells when these were grown on CMC or the control substrate compared to the DEX substrate’s somewhat elongated cells. Either shape means that the cells grow on the surface (elongated cells are often formed under high cell density conditions and generally indicate a favorable surface for specific cell growth, [54]).

**Figure 9:**
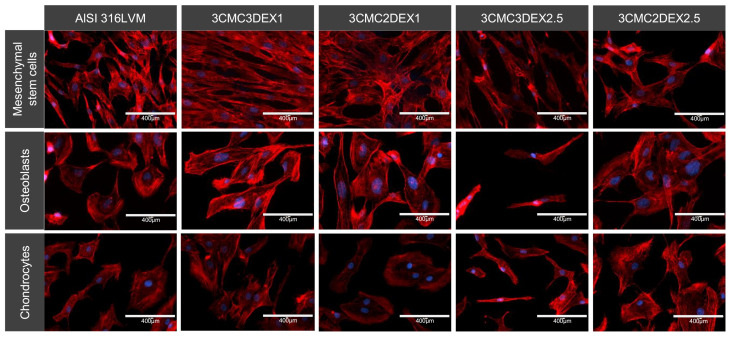
Representative images of the cell morphology and adhesion of different cell types cultured on four different CMC/DEX-coated substrates and controls (AlSI 316LVM) analysed by F-actin staining (red) and visualised with fluorescent microscopy. Cell nuclei were stained with DAPI (blue).

**Figure 10:**
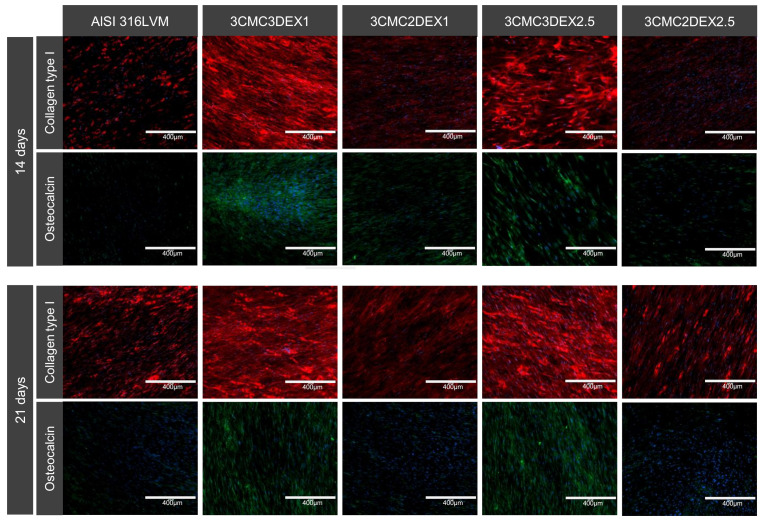
Dexamethasone induces osteogenic differentiation of MSCs. Cells were incubated for 14 and 21 days before immunostaining for osteo-specific markers. Red: collagen type I. Green: osteocalcin. Blue: cell nuclei (DAPI).

Finally, the potential of developed coatings was investigated to induce MSC differentiation. To this end, immunocytochemical analysis of MSC osteogenic and chondrogenic differentiation markers was performed (Figure 9 and Figure S3 in the Supplementary Materials). Cells were cultured on the substrates for 14 and 21 days, followed by the staining for collagen type I and II, osteocalcin, and aggrecan. Collagen type I and osteocalcin indicate an osteogenic differentiation, whereas collagen type II and aggrecan indicate a chondrogenic differentiation. As shown in Figure 10, increased expression of collagen type I and osteocalcin in the cells grown on substrates containing DEX was observed as the top layer compared to substrates containing CMC as the top layer in the case of uncoated controls. These results indicate that the direct contact of MSCs with DEX favors osteogenic differentiation. No formation of the chondrogenic markers was observed under the experimental conditions (Figure S3 of the Supplementary Materials). Furthermore, immunocytochemical analysis of the osteoblasts and chondrocytes (Figure S4 of the Supplementary Materials) showed that both cell types express their specific matrix proteins when grown on CMC/DEX-coated substrates.

In conclusion regarding the obtained immunocytochemical results, it can be stated that the prepared coatings promote the growth of the main cell types (chondrocytes, osteoblasts, and MSCs) present at the target site of orthopedic interventions (e.g., in hip replacements). The latter was confirmed by viability testing, as well as through specific marker production. Furthermore, the samples with DEX as the top-most layer even promoted osteogenic differentiation, which indicated improved osteointegration of these coatings. The latter is in agreement with the literature, where DEX was shown to be able to promote the osteogenic differentiation of MSCs [55]. However, the latter requires controlled drug release, which in this case was achieved by the prepared coatings.

## 4. Conclusions

This study presents the development and characterization of multilayer carboxymethylcellulose (CMC)/dexamethasone (DEX) coatings. DEX was used to achieve an anti-inflammatory effect and improve the osteointegration potential of the coatings through improved osteogenic differentiation of MSCs. To increase the DEX dosage, β‑cyclodextrin was employed, which significantly increases DEX solubility and thus allows higher DEX content in the coating. The coatings were prepared by spin coating alternating CMC and DEX layers on medical-grade stainless steel (AISI 316LVM). Five-layer (3 CMC and 2 DEX) and six-layer (3 CMC and 3 DEX) coatings, with CMC being the first layer in both cases.

Successful layer-by-layer coating formation was confirmed using attenuated total reflectance Fourier transform infrared spectroscopy and atomic force microscopy, which further showed increased surface roughness through coating application. The latter is a favorable feature in terms of cell growth promotion. Furthermore, it was shown that the application of CMC/DEX coatings increases the hydrophilic character of AISI 316LVM. The release of DEX from these coatings followed a zero-order and/or Higuchi model release mechanism. The DEX was released in four stages; the initial “burst” release (in the immersion interval from 0–10 min), followed by fast release (10–30 min), slow release (60–360 min), and the plateau stage (360–4320 min).

Electrochemical measurements were carried out to check the possible influence of the applied coatings on the susceptibility of AISI 316LVM to corrosion. Based on the electrochemical impedance spectroscopy measurements and cyclic polarization curve measurements, it was concluded that the coating application does not significantly influence the general (uniform) and localized corrosion of AISI 316LVM. A similar general corrosion rate and a similar (or even lower) localized corrosion rate can be expected for the coated samples compared with the uncoated samples in human body solutions.

All prepared coatings improved the viability of the most abundant cell types found at the site of common orthopedic interventions (mesenchymal stell cells—MSC, osteoblasts, and chondrocytes) after six days of culturing. Moreover, coatings with DEX at the top-most layer promoted osteogenic differentiation of MSC, indicating their potential to promote osteointegration.

## Figures and Tables

**Table 1:** Measured contact angles.

Sample	Contact Angle [°]
BLANK	83.04 ± 4.07
3CMC3DEX1	4.94 ± 0.22
3CMC2DEX1	4.69 ± 0.21
3CMC3DEX2.5	2.67 ± 0.19
3CMC2DEX2.5	2.32 ± 0.21

**Table 2:** Mathematical drug release models with corresponding plotting parameters.

Model	Plotting
ZERO ORDER	*w* vs. *t*
FIRST ORDER	log *w* vs. *t*
HIXSON-CROWELL	*Q*_max_^1/3^—*Q*_t_^1/3^ vs. *t*
HIGUCHI	*w* vs. *t*^1/2^
KORSMEYER-PEPPAS	*w* vs. log *t*

**Table 3:** Mathematical release model determination by comparing the fitting parameter *R*^2^. The bold underlined values represent the best fit and hence the most suitable model to describe the respective release stages for the four tested samples.

Model	Stage	3CMC3DCF1	3CMC2DCF1	3CMC3DCF2.5	3CMC2DCF2.5
ZERO ORDER	I	**0.9999**	**0.9961**	**0.9980**	**0.9967**
FIRST ORDER		0.9654	0.9518	0.9415	0.9461
HIXSON-CROWELL		0.9837	0.9716	0.9681	0.9691
HIGUCHI		0.9813	0.9929	0.9897	0.9921
KORSMEYER-PEPPAS		0.9235	0.9491	0.9413	0.9469
ZERO ORDER	II	**0.9994**	**0.9999**	0.9982	0.9965
FIRST ORDER		0.9912	0.9931	0.9861	0.9835
HIXSON-CROWELL		0.9950	0.9965	0.9913	0.9889
HIGUCHI		0.9973	0.9957	**0.9989**	**0.9997**
KORSMEYER-PEPPAS *		0.9841	0.9806	0.9885	0.9917
ZERO ORDER	III	**0.9995**	**0.9990**	**0.9998**	**0.9996**
FIRST ORDER		0.9905	0.9863	0.9935	0.9906
HIXSON-CROWELL		0.9950	0.9923	0.9971	0.9950
HIGUCHI		0.9884	0.9908	0.9838	0.9887
KORSMEYER-PEPPAS *		0.9655	0.9834	0.9725	0.9872
ZERO ORDER	IV	**0.9987**	**0.9992**	0.9944	**1.0000**
FIRST ORDER		0.9977	0.9982	0.9921	0.9996
HIXSON-CROWELL		0.9980	0.9986	0.9929	0.9998
HIGUCHI		0.9984	0.9977	**1.0000**	0.9950
KORSMEYER-PEPPAS *		/	/	/	/

* The model is considered valid for only up to 60% of the maximal total of the released drug.

## Data Availability

The data presented in this study are available on request from the corresponding author.

## Supplementary Materials

The following are available online at https://www.mdpi.com/article/10.3390/pharmaceutics13040568/s1, Figure S1: ATR-FTIR spectra for comparison of respective components in the multilayer coatings in solution and pure chemicals (powders), Figure S2: Overlay of ATR-FTIR spectra of β-cyclodextrin (β-CD), dexamethasone (DEX), and DEX+CDaq mixture, showing combined spectral features around 1000 cm^−1^, Figure S3: Immunocytochemical analysis of chondro-specific markers (Collagen type II and aggrecan) in hMSCs after 14 and 21 days of incubation on different CMC/DEX coated and non coated substrates, Figure S4: Immunocytochemical analysis of hFOB and chondrocytes. The expression of chondrocyte and osteocyte-specific markers was evaluated after 7 days of incubation on different CMC/DEX coated and non-coated substrates.
