# Supplementary material for: Dexamethasone-Loaded Bioactive Coatings on Medical Grade Stainless Steel Promote Osteointegration
Source: Pharmaceutics. 2021 Apr 16;13(4):568. doi: 10.3390/pharmaceutics13040568 (PMC8073817; doi:10.3390/pharmaceutics13040568)
Supplement: Supplementary file 1 [file pharmaceutics-13-00568-s001.zip › pharmaceutics-1167956-supplementary.pdf]

pharmaceutics
MDPI

Supplementary Materials: Dexamethasone-Loaded Bioactive Coatings on Medical Grade Stainless Steel Promote Osteointegration

Jan Rožanc, Marko Žižek, Marko Milojević, Uroš Maver and Matjaž Finšgar

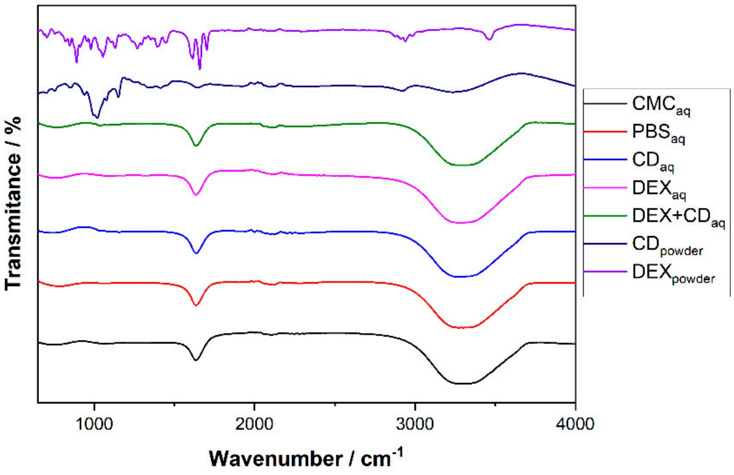

Figure S1. ATR-FTIR spectra for comparison of respective components in the multilayer coatings in solution and pure chemicals (powders).

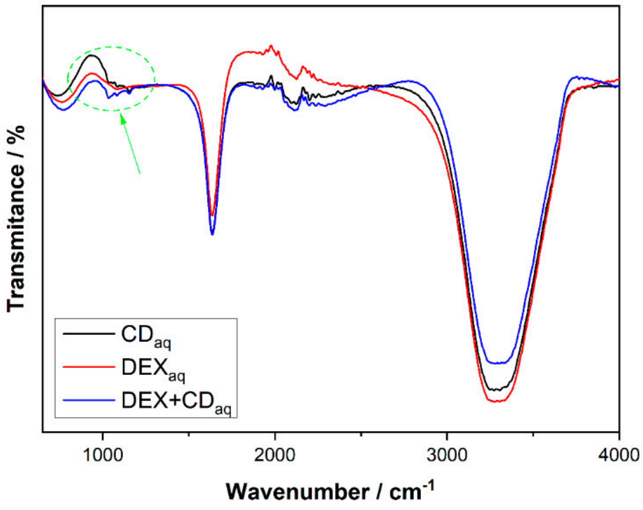

Figure S2. Overlay of ATR-FTIR spectra of β-cyclodextrin (β-CD), dexamethasone (DEX), and DEX + CD_{aq} mixture, showing combined spectral features around 1000 cm^{-1}.

Publisher's Note: MDPI stays neutral with regard to jurisdictional claims in published maps and institutional affiliations.

Copyright: © 2021 by the authors. Submitted for possible open access publication under the terms and conditions of the Creative Commons Attribution (CC BY) license (http://creativecommons.org/licenses/by/4.0/).

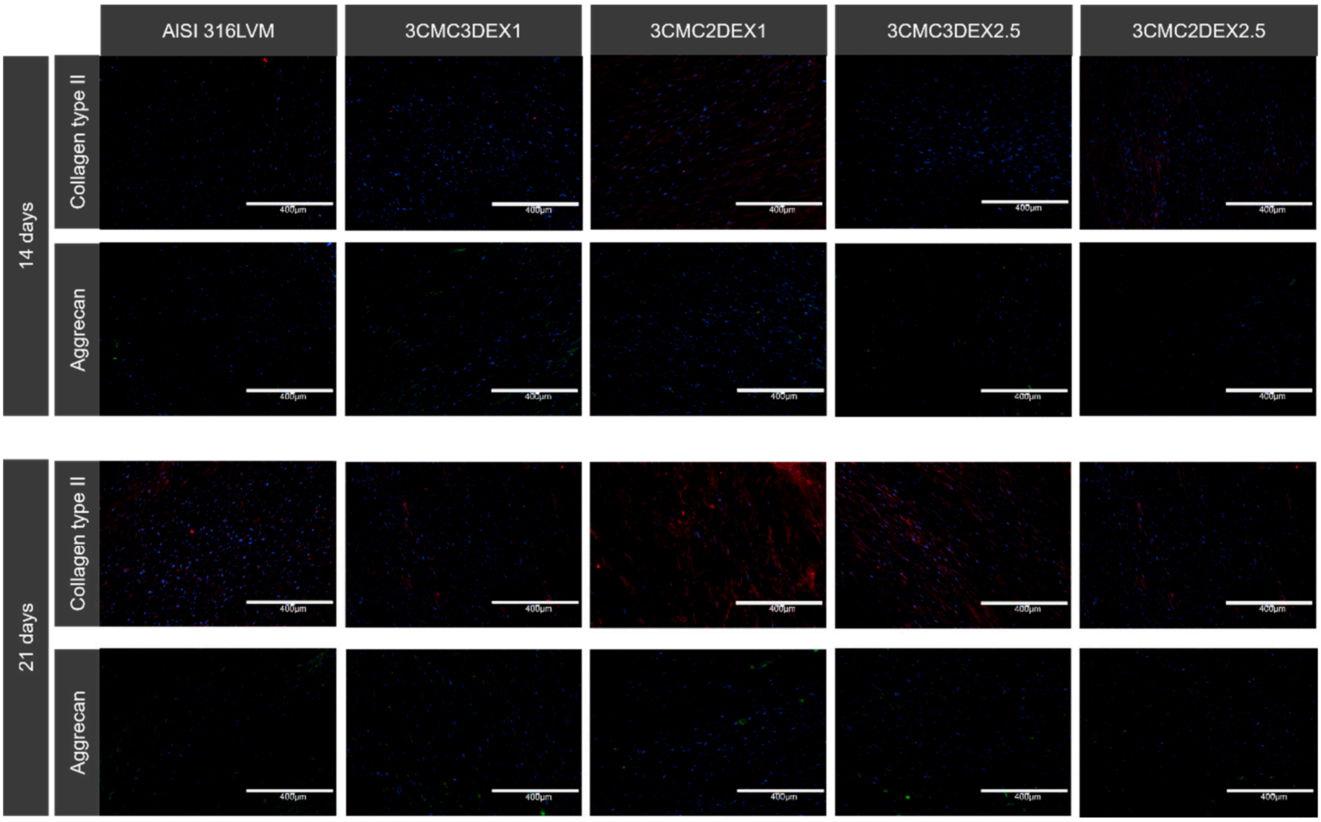

Figure S3. Immunocytochemical analysis of chondro-specific markers (Collagen type II and aggrecan) in hMSCs after 14 and 21 days of incubation on different CMC/DEX coated and non-coated substrates.

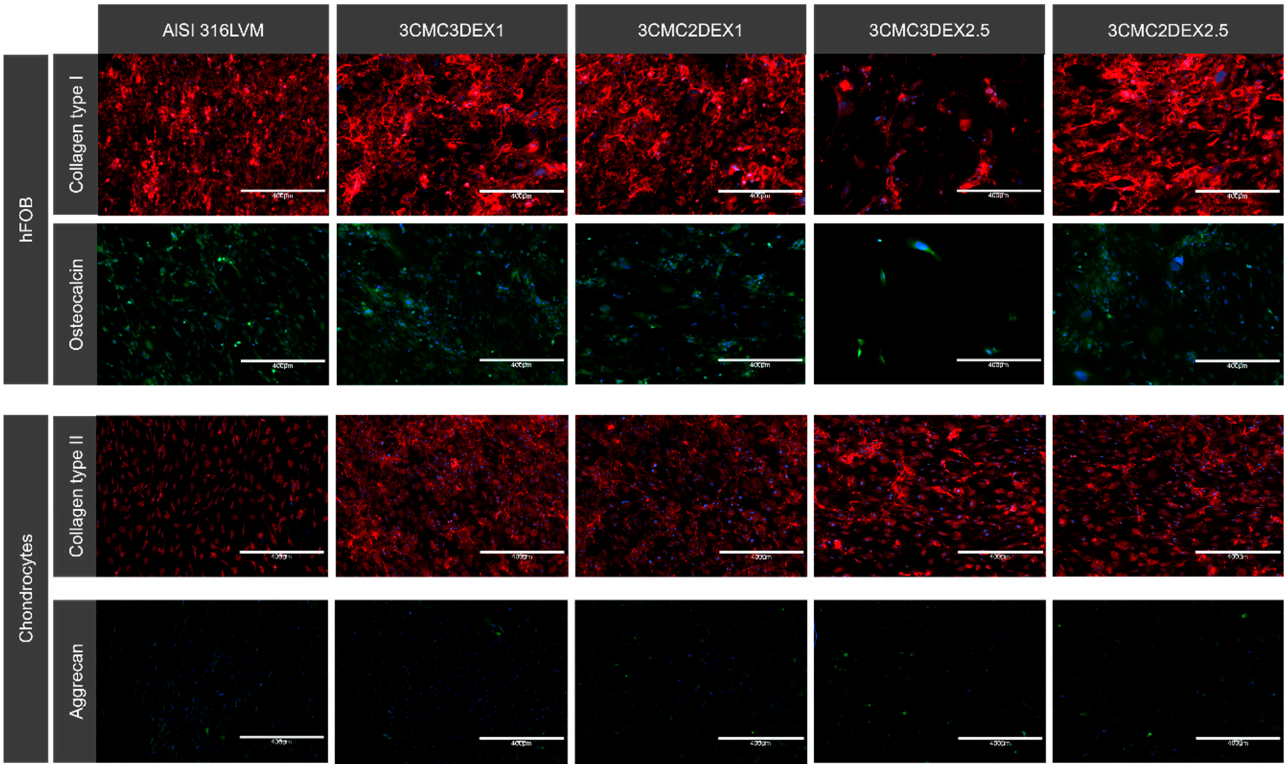

Figure S4. Immunocytochemical analysis of hFOB and chondrocytes. The expression of chondrocyte and osteocyte-specific markers was evaluated after 7 days of incubation on different CMC/DEX coated and non-coated substrates.
